# Intestinal Paracoccidioidomycosis Masquerading as Crohn’s Disease: Diagnostic Pitfalls in a Case Report

**DOI:** 10.7759/cureus.117770

**Published:** 2026-10-11

**Authors:** Ana Cerqueira, Tiago Seco, Joana Moniz, Bárbara Lemos, Mariana Pedrosa

**Affiliations:** 1 Internal Medicine, Unidade Local de Saúde da Região de Leiria, Leiria, PRT

**Keywords:** crohn’s disease, gomori-grocott staining, granulomatous ileocolitis, intestinal paracoccidioidomycosis, lymphadenopathy

## Abstract

Paracoccidioidomycosis (PCM) is a systemic fungal infection caused by *Paracoccidioides* spp., predominantly found in Brazil. It primarily affects the respiratory system, while extrapulmonary involvement is rare. Disseminated PCM in immunocompetent hosts may be associated with exposure burden, host factors, delayed diagnosis, and disease phenotype.

We present the case of a 28-year-old Brazilian man who had been living in Portugal for five months and presented to the emergency department with persistent abdominal pain lasting three weeks. Initial evaluation revealed mesenteric lymph-node conglomerates. During hospitalization, an extensive workup was performed, including screening for active neoplastic disease, immunosuppressive states, and active infection. A fourth-generation HIV combined antigen/antibody test was negative. Immunoglobulin levels were within the normal range (immunoglobulin A 160 mg/dL, immunoglobulin M 200 mg/dL and immunoglobulin G 1500 mg/dL). There was no history of immunosuppressive medication use including steroid or biologic, and no history of diabetes or transplantation. There was also no clinical or imaging evidence of solid or haematological malignancy. Immunophenotyping of peripheral blood and two lymph nodes showed no changes of pathological significance.

Clinical, imaging, endoscopic, and histologic findings suggested Crohn’s disease, although the presentation was atypical because of the extent of painful lymphadenopathy and hepatosplenomegaly. However, Periodic Acid-Schiff (PAS) and Ziehl-Neelsen staining were performed on the initial slides containing granulomas (inguinal lymph node and samples from the ileum and colon, but did not demonstrate evidence of infection at that time). A trial with high-dose corticosteroids was initiated.

Following subsequent deterioration, an axillary lymph-node was excised. Gomori-Grocott (GG) staining, which was only performed at this stage, revealed the “ship’s pilot wheel” appearance. There was no isolation in cultures and serologic and polymerase chain reaction (PCR) testing for *Paracoccidioides* spp. could not be performed at our hospital. Therefore, the diagnosis of paracoccidioidomycosis was established based on histologic morphology, epidemiologic context, and suggestive clinical features. After the diagnosis was established, the initial slides were reviewed and GG staining was added, becoming clear that the fungal forms were already present in all the tested samples, but had gone unrecognized.

Imported fungal infections in non-endemic areas present significant clinical challenges, including delayed diagnosis and limited clinician experience. In Portugal, only one private laboratory can perform PCR and serologic testing for paracoccidioidomycosis. This limitation highlights an important lesson from this case: a diagnosis cannot be made if the disease is not considered. This case may therefore serve as a useful resource for settings with similar diagnostic limitations.

Given the extreme rarity of extrapulmonary PCM, this case highlights the need for a comprehensive diagnostic approach to intestinal granulomatous diseases, particularly in patients from endemic regions, and emphasizes the role of internal medicine in this diagnostic process.

## Introduction

Paracoccidioidomycosis is a systemic fungal infection caused by *Paracoccidioides* species and is endemic to Latin America [1]. Although the lungs are the primary site of involvement, dissemination to other organs, including the gastrointestinal tract, can occur. Gastrointestinal tract is rare (< 1% of cases) and often presents with non-specific symptoms [2]. Although it is more common in immunocompromised patients [3], disseminated disease can also occur in immunocompetent individuals and may be associated with intense exposure or delayed diagnosis.

We present a case of systemic paracoccidioidomycosis with intestinal involvement, highlighting the diagnostic challenges and clinical features of this unusual presentation.

## Case presentation

A 28-year-old Brazilian man who had lived and worked on his parents' farm in the state of Espirito Santo throughout his life, with no relevant medical history or regular medication use, and who had been living in Portugal for five months, presented to the emergency department with persistent abdominal pain, asthenia, anorexia, and weight loss over a three-week period (the BMI at admission was 20 kg/m^2^). Physical examination revealed abdominal pain and distension, along with palpable and painful inguinal, cervical, and axillary lymphadenopathy. Initial workup revealed leukocytosis, an elevated C-reactive protein level (73.5 mg/L), an elevated erythrocyte sedimentation rate (88 mm/h), microcytic hypochromic anemia (hemoglobin 8.7 g/dL), hypoalbuminemia (18 g/L), and mildly elevated liver enzymes (alanine aminotransferase (ALT) 55 U/L, aspartate aminotransferase (AST) 48 U/L, alkaline phosphatase (ALP) 150 U/L and gamma-glutamyl transferase (GGT) 70 U/L). Abdominal CT angiography demonstrated a mesenteric lymph-node conglomerate with the largest lymph nodes measured about 7 cm in maximum diameter, as well as hepatosplenomegaly (Figure 1).

**Figure 1:**
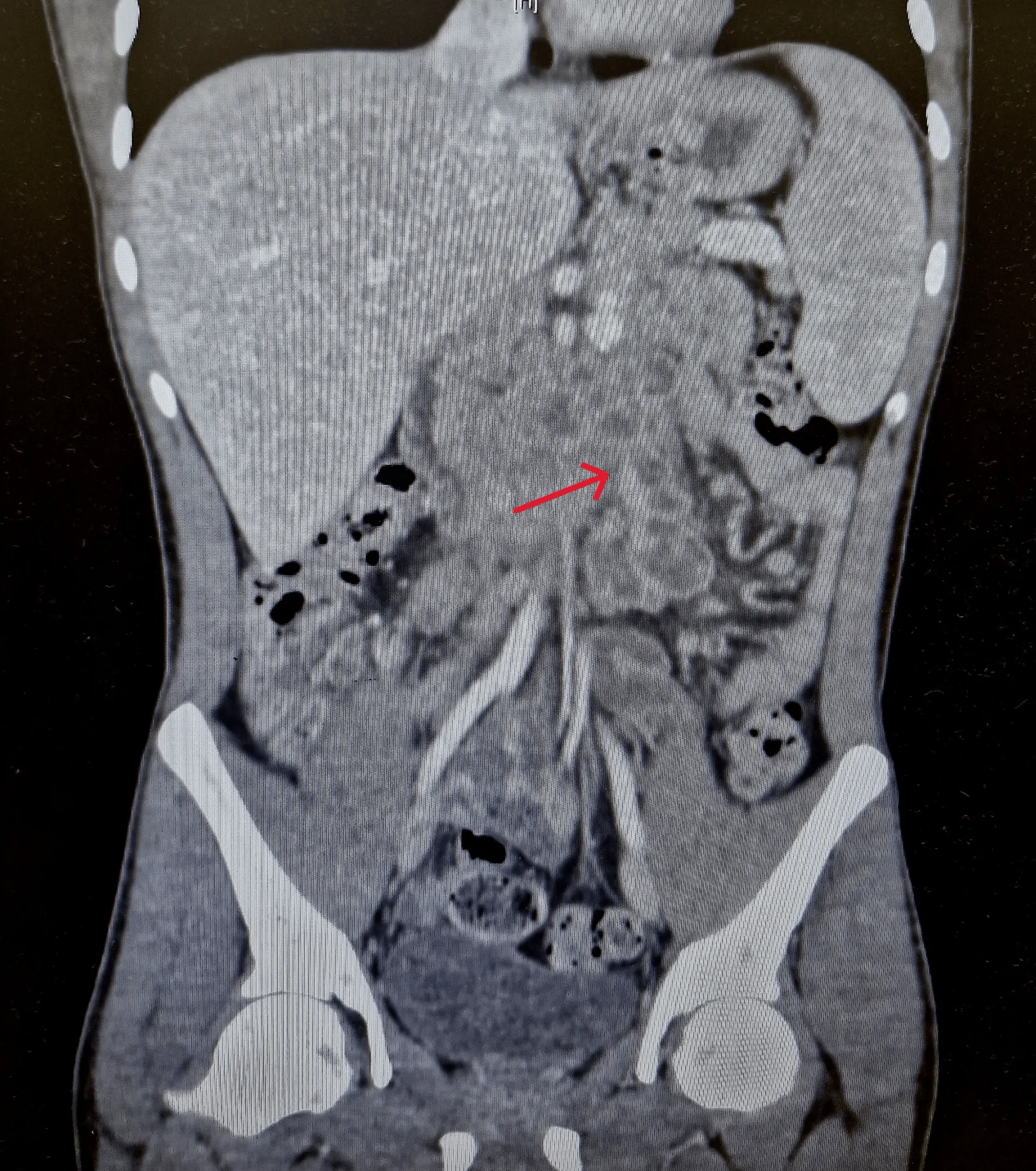
Large intra-abdominal adenopathic conglomerate Abdominal computed tomography scan at emergency department showing hepatosplenomegaly and a marked adenopathic conglomerate in the mesenteric area.

At this stage, he had no history of fever, diarrhoea, or bleeding. He also had no pulmonary symptoms, and the chest X-ray showed no obvious abnormalities. The patient was therefore admitted for further investigation.

During the inpatient stay, extensive infectious and immunological testing revealed no significant abnormalities. Blood cultures were obtained using aerobic, anaerobic, mycobacterial and mycological media; stool samples were collected for routine bacterial stool culture (*Salmonella*, *Shigella*, *Campylobacter*,* Escherichia coli*), *Clostridioides difficile* toxin testing, oval and parasite examination, and fungal stool culture. Samples were also collected from the inguinal, cervical and axillary lymph nodes and several ileocolic segments for culture and polymerase chain reaction (PCR) testing for acid-fast bacilli. Yersinia and PCR testing for HIV and leishmaniasis, as well as serologic and PCR testing for histoplasmosis and PCR testing for Whipple disease using blood and intestinal samples, were also performed. All results came back negative. Active neoplastic disease, including both solid and hematologic, was excluded, and there was no evidence of immunosuppression. Immunophenotyping of peripheral blood and lymph nodes was unremarkable. The patient also underwent a chest CT, which showed no significant abnormalities.

During the first days of hospitalization, sickle cell trait was identified, and the patient was diagnosed with acute appendicitis based on clinical, radiographic, and histological findings. The pathology findings demonstrated desquamation of the appendiceal mucosa into the lumen in association with a polymorphic inflammatory infiltrate and blood. The appendiceal wall showed infiltration by the wall by polymorphonuclear cells and eosinophils, findings consistent with acute suppurative appendicitis. Additionally, numerous epithelioid granulomas were observed adjacent to lymphoid tissue and in the periappendicular soft tissues, with giant cells displaying peripherally arranged nuclei. Grocott staining was subsequently performed on the appendiceal specimens with the identification of fungal organisms, therefore the appendicitis was retrospectively attributed to PCM.

Colonoscopy demonstrated multiple deep, serpiginous ulcers predominantly affecting the terminal ileum and right colon, with an irregular appearance, elevated borders, and areas of edematous mucosa. These findings were compatible with ileocolic Crohn's disease, although they were not diagnostic (Figure 2). 

**Figure 2:**
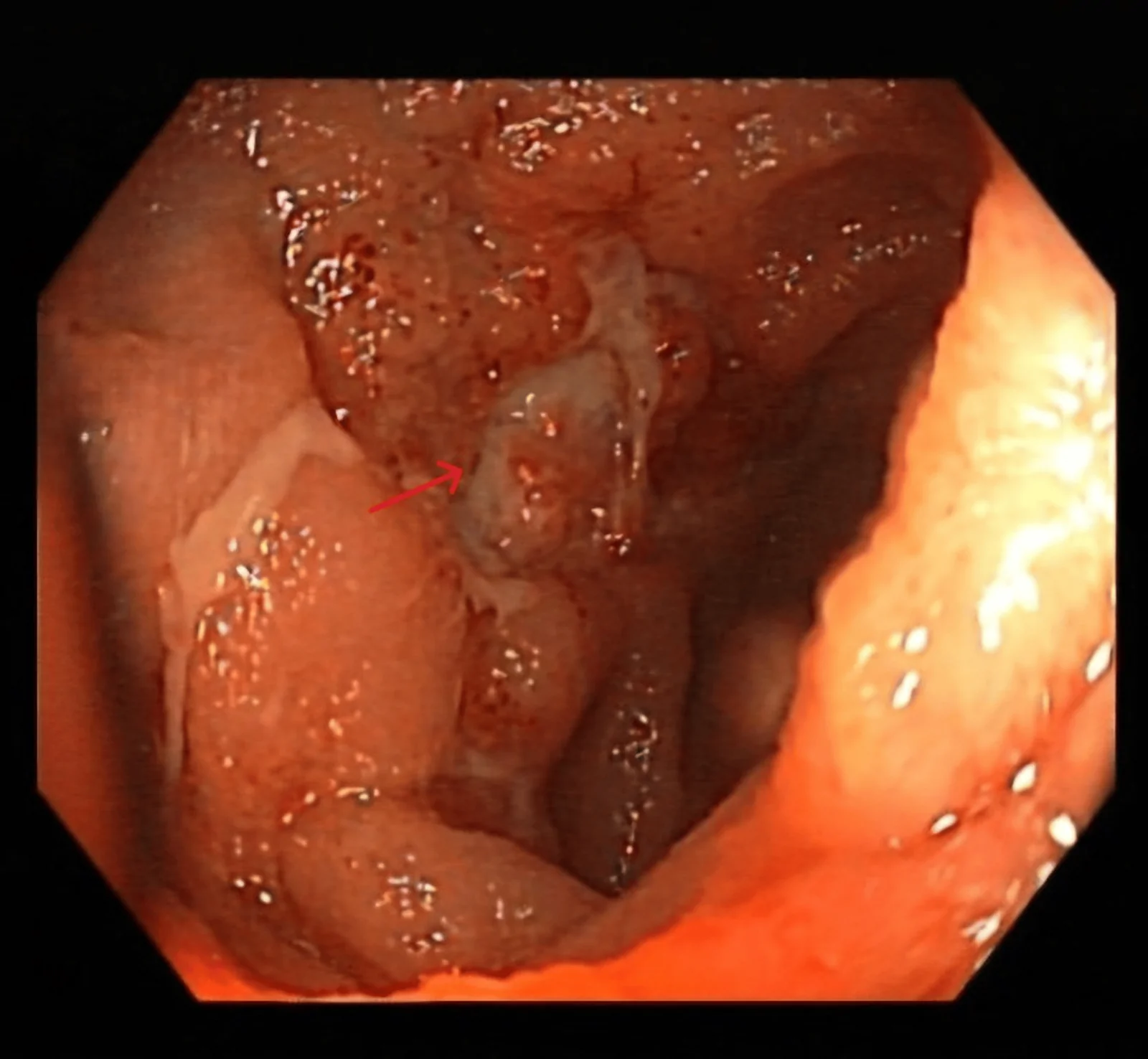
Colonoscopy Terminal ileum with deep, serpiginous ulcerations; valve, cecum, and proximal ascending colon with extensive serpiginous ulcers, some coalescent, and congested mucosa. From the rectum to the ascending colon, numerous pericentimetric aphthous ulcers with an erythematous halo were identified.

MR enterography further revealed scattered bowel-wall thickening without abscesses or fistulas, findings that were also suggestive of inflammatory bowel disease.

Several histologic specimens were collected for further characterization, including inguinal lymph-node, ileal, colonic, and appendiceal tissue samples. All specimens consistently demonstrated a chronic inflammatory infiltrate and numerous scattered non-caseating granulomas. Periodic acid-Schiff (PAS) staining and staining for acid-fast bacilli were negative. At this stage, GG staining had not yet been performed. 

At this point, although no active infectious focus had been identified, the patient had already completed 10 days of empiric broad-spectrum antibiotic and antifungal therapy (intravenous meropenem 1 g 8/8 hours and voriconazole 4 mg/kg every 12 hours) with continued clinical deterioration. 

Given the clinical, laboratory, imaging, and endoscopic evidence, and following a multidisciplinary meeting with colleagues from Gastroenterology and Infectious Diseases, high-dose of corticosteroid therapy was initiated for presumed inflammatory bowel disease (hydrocortisone 300 mg per day intravenously) despite the atypical systemic involvement of this magnitude. By the end of the first week, the patient exhibited marked clinical deterioration, including persistent fever, pneumonia with pleural effusion, anasarca, marked enlargement of disseminated lymphadenopathy with increasing pain, and scattered skin lesions predominantly confined to the head and scalp (Figure 3).

**Figure 3:**
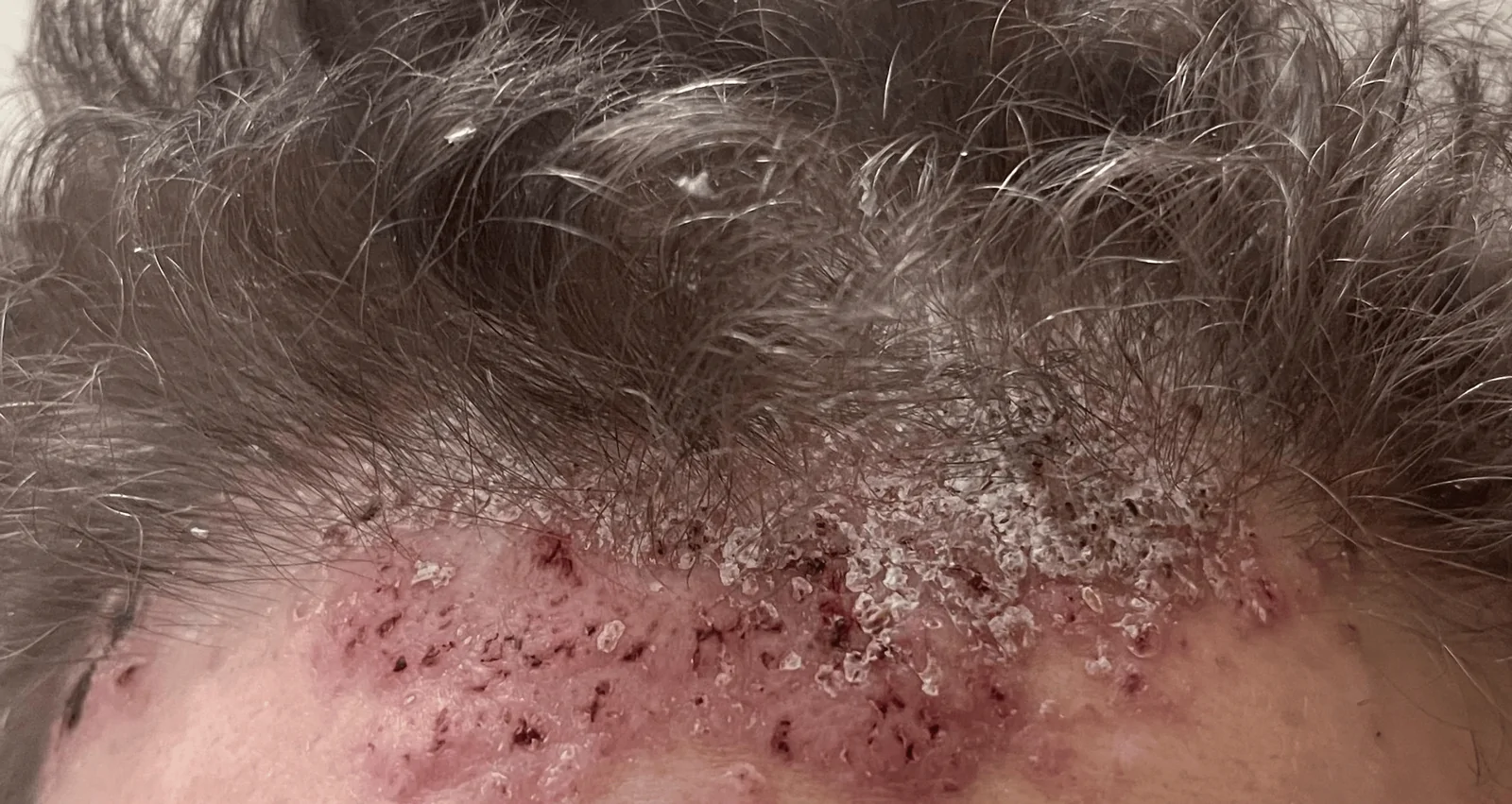
Mycotic skin lesions

These lesions were characterized by irregular borders, scaly appearance, and a tendency to bleed easily. 

A new histologic specimen was obtained by excision of an axillary lymph node, together with a biopsy of the skin lesion. This time, GG staining was performed and was positive for fungi. In addition to the previously described findings, the classic “ship’s pilot wheel” appearance was identified, which added to the epidemiological context and clinical features allowed for a definitive diagnosis of paracoccidioidomycosis (Figure 4).

**Figure 4:**
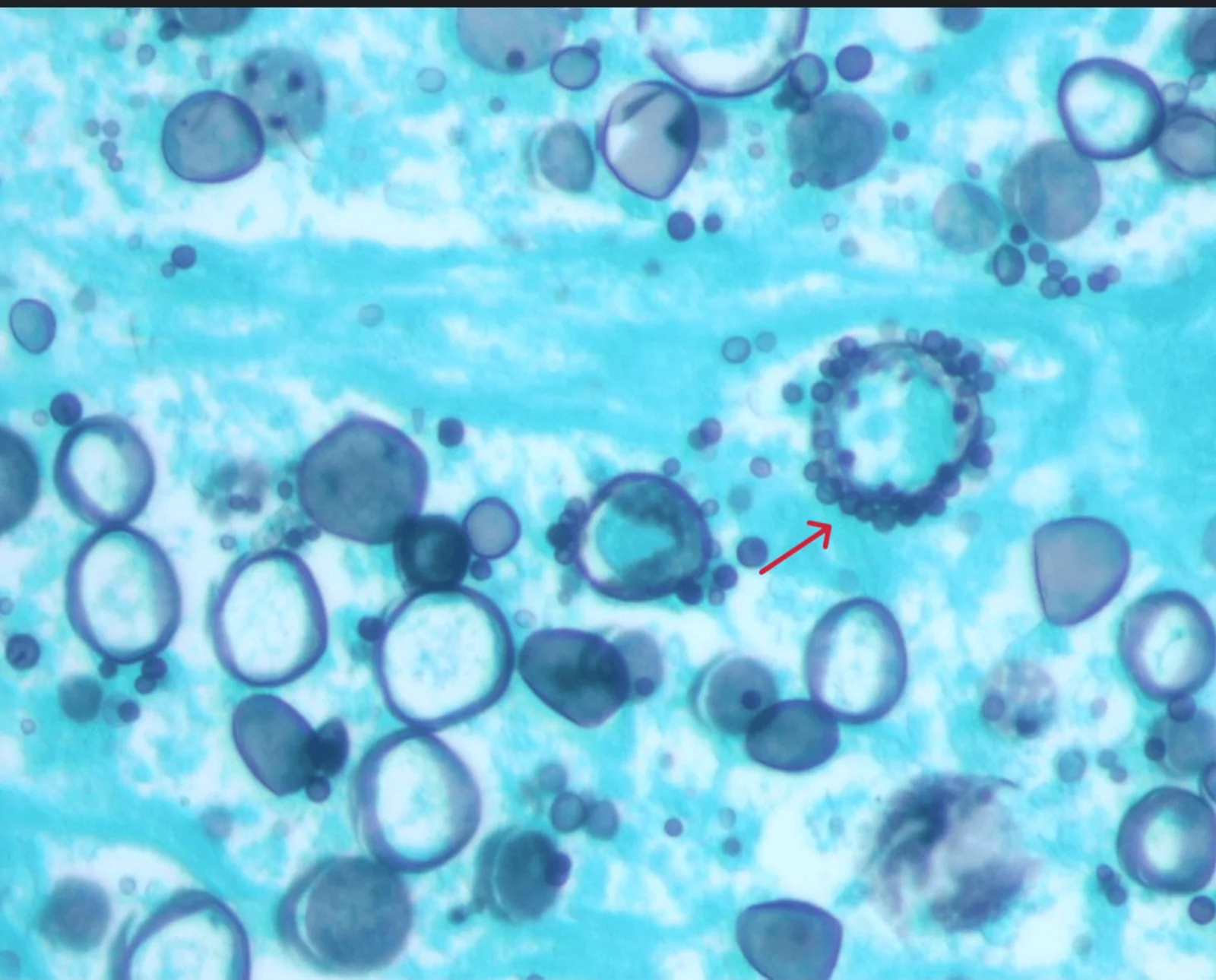
Ship’s pilot wheel on Gomori-Grocott (GG) staining GG staining reveals multiple large, round yeast cells with thick walls, often showing characteristic multiple budding. These morphologic features strongly support the diagnosis of paracoccidioidomycosis.

Treatment was therefore changed to liposomal amphotericin B, which the patient received for approximately one month, resulting in overall clinical improvement. He received a dose of 4 mg/kg/day, which was well tolerated, with occasional need for correction of hypokalaemia. After significant clinical improvement, including sustained apyrexia, absence of abdominal pain, reduction in lymphadenopathy, and a marked decrease in inflammatory markers, and completion of the recommended four-week induction phase with anphotericin B, he was transitioned to maintenance therapy with itraconazole started 200 mg per day, which was continued for an additional month. With appropriate liver biochemical and therapeutic drug monitoring, there was a need for a continuous dose increase.

**Table 1:** Clinical and analytical markers at admission and at the beginning of each treatment option ESR: Erythrocyte sedimentation rate; CRP: C-reactive protein.

	Admission (Day 1)	Corticosteroids (Day 15)	Amphotericin (Day 32)	Itraconazole (Day 64)
ESR (mm)	88	38	58	47
CRP (mg/L)	73.5	267	147	89.5
Albumin (g/L)	18	19	23	27
Hemoglobin (g/dL)	8.7	10.7	7.9	8.6
Ferritin (ng/mL)	712	3500	600	350
Weight (Kg)	61	58	51	54

Despite optimization of measures to improve absorption (proton pump inhibitors and other medications that could impair absorption or interact with itraconazole were previously discontinued) and administration of high-dose of itraconazole (600 mg per day with a meal, taken with cola and fatty foods such as avocado or olive oil), the oral solution proved insufficient because of extensive intestinal involvement by PCM. The altered gastrointestinal motility and mucosal damage led to severe malabsorption, resulting in malnutrition and subtherapeutic drug levels and increasing the risk of treatment failure.

Therapeutic drug monitoring, performed every two weeks, repeatedly demonstrated subtherapeutic levels (the highest value obtained was 0.5 μg per ml, while the intended therapeutic target was 1-4 μg per ml). This was accompanied by worsening of both clinical and laboratory markers. Consequently, at the time of this report, the patient is again being treated with amphotericin B. Pharmacological management remained ongoing, and the conditions for another attempt at oral treatment were still in discussion between the medical team. The available published literature offers limited evidence to support a robust strategy for cases with such significant intestinal involvement. At this point, a joint multidisciplinary strategy was being considered including extending the duration of amphotericin induction, shortening the intervals between itraconazole monitoring, and optimizing nutrition through measures intended to enhance absorption, such as fractionate meals, avoiding foods that interfere with absorption, using liquid or dispersible formulations, gastric acid suppression, being aware of other drug interactions and maintaining adequate good overall nutritional status. Fluconazole, voriconazole and posaconazole are considered alternatives in cases of intolerance, contraindication, or limited access to itraconazole, as well as in complicated or refractory cases; however, they are not considered part of first-line therapy, and clinical experience with these agents remains very limited.

## Discussion

Paracoccidioidomycosis is a systemic mycosis caused by dimorphic fungi of the genus *Paracoccidioides*, mainly *P. brasiliensis* and *P. lutzii*. These fungi exhibit mycelial growth in the environment and convert to yeast forms within the human host [4]. This disease is endemic in Latin America, with the highest incidence reported in Brazil. It predominantly affects men aged 30 to 60 years particularly agricultural workers exposed to contaminated soil [2].

Approximately 95% of infected individuals remain asymptomatic. When symptomatic, the disease manifests in two main clinical forms: acute/subacute (juvenile) and chronic (adult). Infection occurs primarily through inhalation of soil-borne conidia [4]. In the lungs, conidia transform into yeast forms and may disseminate hematogenously and lymphatically to other organs. Intestinal involvement is particularly rare, accounting for <1% of reported cases, and usually results from hematogenous dissemination, although direct ingestion of the fungus may also occur [2,4].

Intestinal PCM may present with nonspecific symptoms such as abdominal pain, diarrhea, weight loss, and fever. Intestinal lesions may mimic inflammatory bowel diseases, particularly Crohn’s disease, with the possible deep ulcers, strictures, and abdominal masses. In even rarer cases, intestinal PCM may represent the initial manifestation of the disease without evident pulmonary involvement or respiratory symptoms, which are commonly recognized and suspected in endemic areas [1,2].

Diagnosis is based on identification of the fungus by culture studies/serology or, alternatively, the simultaneous presence of compatible clinical findings, a suggestive epidemiological context, and histologic specimens demonstrating thick-walled budding yeast forms with multiple buds on GG staining - the classic “ship’s pilot wheel” appearance [5,6].

It is essential to consider intestinal paracoccidioidomycosis in the differential diagnosis of granulomatous ileocolitis, especially in patients with relevant epidemiological background, systemic lymphadenopathy, hepatosplenomegaly, weight loss, and atypical features of Crohn's disease. Fungal stains and endemic mycosis testing should be pursued very early in the diagnostic process to reduce the risk of inappropriate and potentially lethal treatments with corticosteroid or biologic therapies [7,8]. 

Oral itraconazole is the treatment of choice for mild to moderate disease. In disseminated forms or with central nervous system involvement, an induction phase with liposomal amphotericin B 3-5 mg/kg/day is recommended and should be continued until clinical improvement (usually 2-4 weeks), followed by maintenance therapy. Itraconazole should be started at 200 mg/day and subsequently titrated based on plasma levels and clinical response.

Relapses are common, and each relapse can require a new induction phase with amphotericin B, although there may be an increased risk of resistance development [1]. In our patient, the documented subtherapeutic itraconazole concentration provides a plausible explanation for clinical worsening without invoking acquired antifungal resistance, at least at this stage.

Treatment is prolonged and, particularly in severe cases, may last up to 12 to 18 months. In this particular case, given the extent of intestinal involvement, antifungal therapy may have failed to achieve therapeutic blood levels, resulting in an insufficient clinical response and increasing the risk of disease progression, relapse, or development of resistance [9]. Additionally, prolonged use of intravenous formulations may be necessary, increasing the risk of adverse effects and complicating outpatient management. Follow-up must include frequent monitoring of drug plasma levels and close surveillance of clinical evolution [10].

## Conclusions

Intestinal paracoccidioidomycosis may clinically and endoscopically mimic Crohn’s disease, making the initial diagnosis particularly challenging, especially in hospitals with teams and support services that have limited experience with this type of patients. The case supports the need to exclude endemic fungal infections before corticosteroid or biologic therapy in granulomatous ileocolitis, systemic lymphadenopathy and relevant epidemiology. Fungal stains, such as Gomori-Grocott, should be requested early in patients presenting with this constellation of clinical and epidemiological findings.

More than two months after the diagnosis, our patient remains on antifungal therapy with anphotericin B because of poor intestinal absorption. A previous switch to itraconazole failed because therapeutic drug levels were not achieved, resulting in clinical worsening. Future challenges include identifying a set of clinical/analytical/imageological markers of treatment response that can predict adequate itraconazole absorption and help determine the timing of a new attempt at oral therapy, as well as preventing and monitoring potential complications associated with prolonged antifungal therapy.

## References

[1] Shikanai-Yasuda MA, Mendes RP, Colombo AL (2017). Brazilian guidelines for the clinical management of paracoccidioidomycosis. Rev Soc Bras Med Trop.

[2] Giron F, Vanegas M, Rodriguez LM, Hernandez-Santamaria V, Rey Chaves CE, Ortega J (2022). Intestinal paracoccidioidomycosis: case report and literature review. Int J Surg Case Rep.

[3] Felipe CR, Silva AD, Moreira Guimarães Penido MG (2021). Disseminated paracoccidioidomycosis in a kidney transplant recipient. Cureus.

[4] Bellissimo-Rodrigues F, Bollela VR, Da Fonseca BA, Martinez R (2013). Endemic paracoccidioidomycosis: relationship between clinical presentation and patients' demographic features. Med Mycol.

[5] Queiroz-Telles F, Fahal AH, Falci DR, Caceres DH, Chiller T, Pasqualotto AC (2017). Neglected endemic mycoses. Lancet Infect Dis.

[6] Ambrosio AVA, Camelo CCS, Barbosa CV (2014). Paracoccidioidomycosis disease (Lutz-Splendore-Almeida disease): additional workup, differential diagnosis, cure control (in Portuguese). Rev Med Minas Gerais.

[7] Restrepo-Moreno A (2003). Paracoccidioidomycosis. Clinical Mycology.

[8] Andrade IL, Saad BA, Bertucci AA (2025). Cecal appendicitis as a rare manifestation of paracoccidioidomycosis: a case report and systematic review of the literature. J Venom Anim Toxins Incl Trop Dis.

[9] da Cruz ER, Forno AD, Pacheco SA (2021). Intestinal paracoccidioidomycosis: case report and systematic review. Braz J Infect Dis.

[10] Bocca AL, Amaral AC, Teixeira MM, Sato PK, Shikanai-Yasuda MA, Soares Felipe MS (2013). Paracoccidioidomycosis: eco-epidemiology, taxonomy and clinical and therapeutic issues. Future Microbiol.

